# Mycotoxin Exposure and Related Diseases

**DOI:** 10.3390/toxins12030172

**Published:** 2020-03-11

**Authors:** Ricardo Assunção, Susana Viegas

**Affiliations:** 1 Food and Nutrition Department, National Institute of Health Dr. Ricardo Jorge, Avenida Padre Cruz, 1649-016 Lisboa, Portugal; 2 CESAM, Centre for Environmental and Marine Studies, University of Aveiro, Campus Universitário de Santiago, 3810-193 Aveiro, Portugal; 3 NOVA National School of Public Health, Public Health Research Centre, Universidade NOVA de Lisboa, 1600-560 Lisbon, Portugal; 4 Comprehensive Health Research Center (CHRC), 1150-090 Lisbon, Portugal; 5 H&TRC-Health & Technology Research Center, ESTeSL-Escola Superior de Tecnologia da Saúde, Instituto Politécnico de Lisboa, 1990-096 Lisbon, Portugal

Mycotoxins are considered the most frequently occurring natural contaminants in the diet of humans and animals. These toxic secondary metabolites of low molecular weight and very stable compounds are produced by different genera of filamentous fungi that infect susceptible plants throughout the world [1,2]. Considering their particular vulnerability to fungi contamination, crops represent a special concern under mycotoxins context. Most fungal strains produce more than one type of mycotoxin, therefore, co-contamination of agricultural products with multiple mycotoxins is frequently observed, and the need to consider this aspect in the risk assessment process has been emphasized [3,4].

Animals can be exposed to mycotoxins through the consumption of contaminated feed, subsequently entering into the food chain, and thus constituting a source of exposure to humans [5]. Regarding human exposure, in addition to the dietary source, the workplace environment can also represent an exposure source. Dust containing mycotoxins is released during regular tasks involving high exposure to organic dust, such as storage work, loading, handling, or milling contaminated materials (grain, waste, and feed), and other tasks such as caring for animals in animal husbandry settings [5,6,7,8,9,10,11,12,13,14,15].

The establishment of a disease is largely influenced by the magnitude of a given exposure. Consequently, every effort that contributes to properly characterizing the risk associated with human exposure assumes particular relevance.

The present Special Issue aims to shed light on the different perspectives of mycotoxins exposure and their implications for the establishment of a disease. The gathered studies include several important findings focusing on different perspectives and clues about the impact of human and animal exposure to mycotoxins. A broad spectrum of mycotoxins-related issues associated with mycotoxin exposure and related diseases are covered in the present Special Issue.

The detection and quantification of mycotoxins in food and feed, as an important aspect in the exposure characterization process, is focused on in two studies. An innovative detection methodology of aflatoxin M1 (AFM1) in milk using interferometric biosensors has been developed, demonstrating that viable solutions for lab-on-chip devices for food safety analyses are possible and reliable [16]. Data on the individual and combined occurrence of *Fusarium* mycotoxins and ochratoxin A (OTA) in feedstuffs in Costa Rica were collected, highlighting the implications for all stakeholders linked to the feed industry as well as the potential measures that can be considered for the management of mycotoxins in animal production [17].

The risk assessment of human exposure to mycotoxins is also considered, applying different approaches for the general population [18] or to specific populations such as children [19] or swine production workers [6]. Regarding these studies, human biomonitoring strategies, as a direct measure of internal exposure, are considered [6,18]. The exposure to mycoestrogens, namely zearalenone (ZEN) and alternariol, was estimated through data modeling, assessing the burden regarding endocrine disruption [18]. The workplace environment also represents an important exposure source to mycotoxins, namely, in swine production [6]. Exposure of children to mycotoxins in Vietnam were assessed and revealed a high risk associated with high levels of exposure and exceedance of toxicological reference levels [19]. In order to clarify the potential role of the mycotoxin HT-2 in the Kashin–Beck disease, an in vitro approach using immortalized human chondrocyte cell line, C-28/I2, is considered [20]. The study reports a potentially negative effect led by HT-2 exposure and highlights the importance of future studies to provide a better understanding of the mechanism of HT-2 toxin cytotoxicity.

In addition to the human studies, several papers examine the role of mycotoxins in the establishment and/or development of different health effects in animals [21,22,23,24]. Interference of mycotoxins exposure in the gut microbiome and immunity are evaluated in gilts, turkeys, and rats [22,23,24]. In pre-pubertal gilts, a minimal anticipated biological effect level (MABEL) dose of ZEN stimulated the growth of specific strains of intestinal microbiota [22]. In turkeys, the effects of aflatoxin B1 (AFB1) on the gastro-intestinal tract are investigated and show that, in addition to the hepatic transcriptome, animal resistance to this mycotoxin occurs in organ systems outside the liver [23]. In rats, and also focusing on the effects of AFB1, the findings suggest that AFB1 can alter the gut microbiota composition and that *Lactobacillus casei* Shirota can reduce the AFB1-induced dissimilarities in the gut microbiota profile [24]. Hepatoxicity associated with the exposure of piglets to fumonisin B1 (FB1) is also studied [21]. Results show that histology, cellular enzyme leakage, and hepatocellular membrane lipid fatty acid profile are affected after an exposure of 10 days to FB1.

Recognizing the potential negative impact associated to animal exposure to mycotoxins, the application of appropriate mitigation measures is also studied. The use of the yeast cell wall extract (YCWE) in chickens [25] and a novel modified hydrated sodium calcium aluminosilicate (HSCAS) in chicks [26] as adsorbents to mycotoxins are investigated. First, data showed a decrease of up to 30% in OTA deposits in the liver of broilers fed both OTA and YCWE [25]. Second, the results suggest that the modified HSCAS adsorbent can be used against T-2 toxin-induced toxicity in growth performance, nutrient digestibility, and hepatic and small intestinal injuries in chicks [26].

Altogether, and especially under an expected climate change scenario, which considers mycotoxins as an important driver of health consequences, the present Special Issue contributes with significant and impactful research that supports the anticipation of potential consequences of the exposure of humans and animals to mycotoxins, future risk assessments, and the establishment of preventive measures.
